# Psychiatric disorders risk in patients with iron deficiency anemia and association with iron supplementation medications: a nationwide database analysis

**DOI:** 10.1186/s12888-020-02621-0

**Published:** 2020-05-11

**Authors:** Herng-Sheng Lee, Hsin-Hao Chao, Wan-Ting Huang, Solomon Chih-Cheng Chen, Hsin-Yi Yang

**Affiliations:** 1 Department of Pathology and Laboratory Medicine, Kaohsiung Veterans General Hospital, Kaohsiung, 813 Taiwan; 2 Department of Psychiatry, Ditmanson Medical Foundation Chia-Yi Christian Hospital, Chiayi, Taiwan Chia-Yi City, 600 Taiwan; 3 Clinical Medicine Research Center, Ditmanson Medical Foundation Chia-Yi Christian Hospital, No. 539, Zhongxiao Rd., East District, Chia-Yi City, Taiwan 60002; 4 Department of Pediatrics, School of Medicine, College of Medicine, Taipei Medical University, Taipei, 110 Taiwan; 5 Department of Pediatrics, School of Medicine, College of Medicine, Kaohsiung Medical University, Kaohsiung, 807 Taiwan

**Keywords:** Iron deficiency anemia, Psychiatric disorders, Iron supplementation, Gender difference

## Abstract

**Background:**

It has been shown that iron deficiency anemia (IDA) is associated with psychosocial consequences and psychiatric morbidity. However, the association between adults with IDA and psychiatric disorders has not been clarified. The purpose of this study was to investigate the psychiatric disorder morbidity of an IDA group in comparison with a non-IDA group and to examine the risk of psychiatric disorders in IDA patients treated with iron supplementation.

**Methods:**

All study subjects were 20 years of age or over with newly diagnosed IDA enrolled in the Taiwan National Health Insurance Database from 2000 to 2012. We matched IDA and non-IDA subjects according to age and gender in a 1:2 ratio. Our primary outcome was diagnosis of psychiatric disorders and the patients were monitored until the end of 2013. A multivariate Cox proportional hazards regression model was used to explore the risk of psychiatric disorders in patients with IDA after adjustment for confounders, including demographic characteristics and comorbidities.

**Results:**

The adjusted hazard ratios (aHRs) of psychiatric disorders was 1.52 (95% CI = 1.45–1.59) in the IDA group compared with the non-IDA group. Among the different types of psychiatric disorders, the IDA group was associated with significantly higher incidence and risks of anxiety disorders, depression, sleep disorders, and psychotic disorders (*p* <  0.05). Furthermore, iron supplementation in IDA subjects was associated with a significantly lower risk of psychiatric disorders compared to non-iron supplementation in IDA patients.

**Conclusions:**

Our study indicates that IDA subjects had an increased risk of psychiatric disorders, regardless of other confounders. In IDA patients, iron supplementation was associated with a decreased risk of psychiatric disorders. Moreover, IDA patients receiving iron supplementation also had a lower risk of sleep disorders.

## Background

Iron deficiency is one of the most common nutritional deficiencies worldwide, affecting more than two billion people [1]. Iron, an indispensable nutritional element for every living organism, is essential for numerous important functions, such as transport of oxygen, cellular respiration, immune function, neurotransmitter metabolism and DNA synthesis [2, 3]. The definition of iron deficiency is a shortage in the total content of iron in the body, which can lead to anemia as well as other health problems including: unusual fatigue [4], headaches and dizziness [5], restless legs [6], impaired immune function [7], pica [8], etc. Furthermore, iron deficiency anemia (IDA) occurs when iron deficiency is sufficiently severe to impair erythropoiesis, contributing to the development of anemia.

An accumulating body of evidence currently indicates that iron has an important role in neurologic function and development. IDA gives rise to poor myelination in the brain and impairment of monoamine metabolism [9]. Current literature indicates that brain iron deficiency influences neurotransmitter (glutamate and γ-aminobutyric acid (GABA)) homeostasis, which causes deficits in memory, learning, and behavior, as well as emotional and psychological problems [10]. In addition, previous research has found that anemia patients were more prevalent among those with cognitive derangement and neurological symptoms [11].

There is growing evidence that IDA is associated with psychosocial consequences, including adverse psychomotor function, reduced work capacity [12], delayed socioemotional development [13], and psychiatric morbidity, including anxiety disorders [14], depression [15], bipolar disorders [14], sleep disorders [16] and restless legs syndrome (RLS) [17]. However, some studies have concluded that there is no association between IDA and psychotic disorders. A cohort study reported no increased risk of cognitive decline in anemia patients [18]. Yi et al. [19] and Millingen et al. [20] showed no association between IDA and depression. This inconsistency may be due to heterogeneous study designs, sample selection criteria, or ethnic differences. These studies also tended to be small scale, and of cross-sectional or case-control design. Therefore, we used a population-based cohort analysis to investigate the psychiatric disorder morbidity of an IDA group in comparison with a non-IDA group and to examine the risk of psychiatric disorders in IDA patients treated with iron supplementation.

## Methods

### Data sources

This retrospective population-based cohort study used the Longitudinal Health Insurance Database 2005 (LHID 2005) released by the Taiwan National Health Research Institutes (NHRI) for research purposes. The National Health Insurance (NHI) Program implemented on March 1, 1995, covers more than 99% of Taiwan’s population of 23.74 million population. The LHID 2005 consists of a random sample of 1 million, and includes demographic data of enrollees; service records and expenditure claims from outpatient, inpatient, and ambulatory care; and data associated with contracted pharmacies for reimbursement purposes. The International Classification of Disease, 9th Revision, Clinical Modification (ICD-9-CM) codes was used to identify diseases in this study. The accuracy of diagnoses in the NHIRD has been verified in previous articles [21–23]. This study was approved by the Institutional Review Board of the Ditmanson Medical Foundation, Chia-Yi Christian Hospital, Taiwan (CYCH-IRB No: 2018078).

### Study population

We conducted a retrospective cohort study covering the period from January 1, 2000 to December 31, 2013. We selected subjects 20 years of age or over with a first diagnosis of IDA (ICD-9-CM: 280) from the LHID 2005 between January 1, 2000 and December 31, 2012. Excluded were patients diagnosed with anxiety disorders, depression, psychotic disorders, bipolar disorders, sleep disorders, or RLS before 2000, or before their first visit for IDA. In order to increase the validity of IDA diagnoses, this study only included cases that had at least two diagnoses of IDA in their medical claims prior to their index date as IDA cases. Supplementary iron medication data were also collected. Detailed iron supplementation classification is shown in Supplementary Data, Table S1. Patients who had taken any oral iron between the investigation follow-up periods were defined as iron users; the remaining subjects were defined as iron non-users. Individuals with missing data and those who were diagnosed without blood tests were excluded. Moreover, on the basis of the clinical guidelines and health insurance regulations of the NHI, patients suspected of having IDA might receive a diagnosis of unspecified anemia (ICD-9-CM: 285) on their first visit. However, in order to confirm the diagnosis of IDA, patients underwent laboratory testing for decreased serum iron and ferritin, and increased total iron binding capacity. We retrieved the non-IDA subjects for the comparison cohort from the remaining insured people among the LHID 2005. Individuals in the comparison cohort were individually matched with those in the IDA cohort at a 2:1 ratio based on age, sex, index year, and the year of IDA diagnosis. Individuals with a diagnosis of psychiatric disorders prior to the index date were excluded. A total of 38,794 non-IDA subjects were included in this study.

### Main outcome

Patients in both the IDA and non-IDA groups were followed up from the index date until the end of December 31, 2013, or until one of the following events occurred: diagnosis with psychiatric disorder, including anxiety disorders (ICD-9-CM: 300), depression (ICD-9-CM: 296.2–296.3, 300.4 and 311), psychotic disorders (ICD-9-CM: 295 and 297–298), bipolar disorders (ICD-9-CM: 296.0, 296.4–296.8), sleep disorders (ICD-9-CM: 307.4 and 780.5), and RLS (ICD-9-CM: 333.90 and 333.99), withdrawal from the NHI program, or death, whichever came first. Moreover, anxiety disorders, depression, psychotic disorders, bipolar disorders, sleep disorders, and RLS were also extracted as outcome variables of interest, separately. As with the main outcome, all of the subjects were also followed until withdrawal from insurance, occurrence of events, or until December 31, 2013.

### Baseline characteristics and comorbidities

The general characteristics of individuals were age, gender, and insurable salary (in New Taiwan Dollars [NT$]; < 19,100, 19,100 – 41,999, ≥ 42,000). The present study used the urbanization stratification of Taiwan townships developed at Taiwan’s NHRIs. This index was derived from a cluster analysis of five indicators: population density, percentage of population with college or greater educational level, percentage of population aged 65 years or over, percentage of population working in agriculture, and density of physicians per 100,000 people. The 368 townships in Taiwan were classified into seven levels of urbanization except for the isolated isles in Kinmen and Lienchiang counties. We further classified the urbanization levels as urban (levels 1 and 2), suburban (levels 3 and 4), rural (levels 5–7) and the isolated isles as remote areas [24]. The covariates of comorbidities that were selected in this study included hypertension (ICD-9-CM: 401–405), diabetes mellitus (DM, ICD-9-CM: 250), dyslipidemia (ICD-9-CM: 272), hyperthyroidism (ICD-9-CM: 242), hypothyroidism (ICD-9-CM: 244), chronic obstructive pulmonary disease (COPD, ICD-9-CM: 490–496), stroke (ICD-9-CM: 430–438), coronary artery disease (CAD, ICD-9-CM: 410–414), chronic kidney disease (CKD, ICD-9-CM: 585) and liver cirrhosis (ICD-9-CM: 571.2, 571.5, and 571.6).

### Statistical analysis

Demographic characteristics were expressed using means and standard deviations for continuous variables, presented as numbers and percentages for categorical variables. The differences in continuous variables were estimated using t-tests, and differences between categorical variables were analyzed using the chi-square test or Fisher exact test, as appropriate. The incidence rate was calculated as the number of first diagnoses of psychiatric disorders per 1000 person-years. Univariate and multivariate Cox proportional hazards models were used to calculate hazard ratios (HRs) and 95% confidence interval (CI) for developing outcomes (including overall events and anxiety disorders, depression, bipolar disorders, sleep disorders, RLS, and psychotic disorders, respectively). Multivariate Cox proportional hazards models were used to explore the associations between IDA and risk of psychiatric disorders, controlling for age, gender, and medical comorbidities. The Kaplan–Meier method and log-rank test were used to estimate the cumulative risks of psychiatric disorders between the IDA and non-IDA groups. A 2-tailed *p* <  0.05 was considered significant. The SPSS for Windows version 21.0 (IBM, Armonk, NY, USA) was used for the statistical analysis of the results. Statistical graphs were plotted with R version 3.5.1, with the KMsurv, survfit and survival packages. Stata statistical software (version 15; StataCorp, College Station, TX, USA) was used to calculate the power. The power for survival data calculation was estimated with the Stata command stpower log-rank and the set up conditions were a sample of at least 19,397 patients, an effect size of 1.50 (expressed as an HR), and an α of 0.05 with a 2-sided test. The statistical power was estimated to be more than 99% and would be able to detect any significant difference in the two groups.

## Results

### Baseline characteristics of the IDA and non-IDA groups

**Fig. 1:**
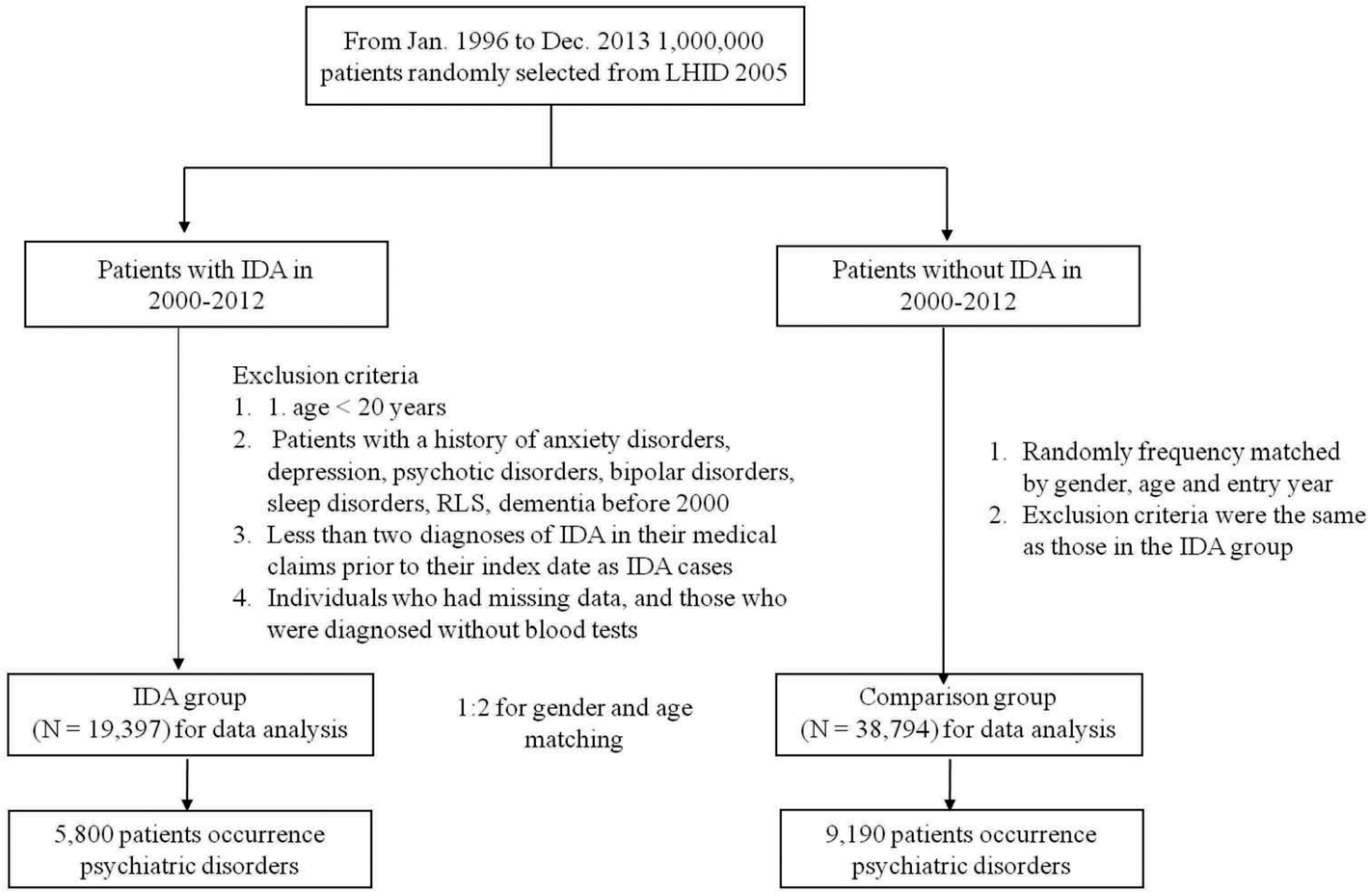
Flow diagram of the present study from the NHIRD

In total, we enrolled 19,397 IDA patients and 38,794 controls. The flow chart for selecting the study population is shown in Fig. 1.The demographic characteristics and comorbidities of the study population are presented in Table 1. The values from IDA and non-IDA groups regarding age (49.08 ± 17.54) and gender (women: 76.77%) are equal, owing to subjects being matched. The IDA patients had a higher prevalence of listed comorbidities and iron supplementation rate than the non-IDA group (*p* <  0.05). In addition, there was a significant difference in the income level and living area (*p* <  0.05) between these two groups.

**Table 1:** Baseline demographic factors and comorbidity of study participants according to IDA

	IDA Group	Non-IDA Group	*p-*value
	*N* = 19,397	*N* = 38,794	
Age	49.08 (17.54)	49.08 (17.54)	0.999
≦ 50	12,024 (61.99)	24,069 (62.04)	
> 50	7373 (38.01)	14,725 (37.96)	
Gender			1.000
Female	14,891 (76.77)	29,782 (76.77)	
Male	4506 (23.23)	9012 (23.23)	
Income level			< 0.001
Low	9366 (48.29)	19,247 (49.61)	
Intermediate	8604 (44.36)	16,523 (42.59)	
High	1427 (7.36)	3024 (7.80)	
Comorbidity			
Hypertension	4991 (25.73)	7874 (20.30)	< 0.001
DM	2719 (14.02)	3529 (9.10)	< 0.001
Dyslipidemia	2822 (14.55)	4435 (11.43)	< 0.001
Hyperthyroidism	551 (2.84)	752 (1.94)	< 0.001
Hypothyroidism	199 (1.03)	257 (0.66)	< 0.001
COPD	3618 (18.65)	5606 (14.45)	< 0.001
Stroke	1642 (8.47)	2235 (5.76)	< 0.001
CAD	2490 (12.84)	3594 (9.26)	< 0.001
CKD	888 (4.58)	412 (1.06)	< 0.001
Cirrhosis	566 (2.92)	216 (0.56)	< 0.001
Iron supplementation	12,450 (64.19)	1551 (4.00)	< 0.001
Area			< 0.001
Urban	11,393 (58.74)	23,754 (61.23)	
Suburban	5974 (30.80)	11,479 (29.59)	
Rural	1286 (6.63)	2216 (5.71)	
Remote area	744 (3.84)	1345 (3.47)	

Data are presented as mean ± SD or number (percentage, %). *DM* Diabetes mellitus, *CAD* Coronary artery disease, *CKD* Chronic kidney disease, *COPD* Chronic Obstructive Pulmonary Disease

### Risk factors for psychiatric disorders in the IDA group

**Fig. 2:**
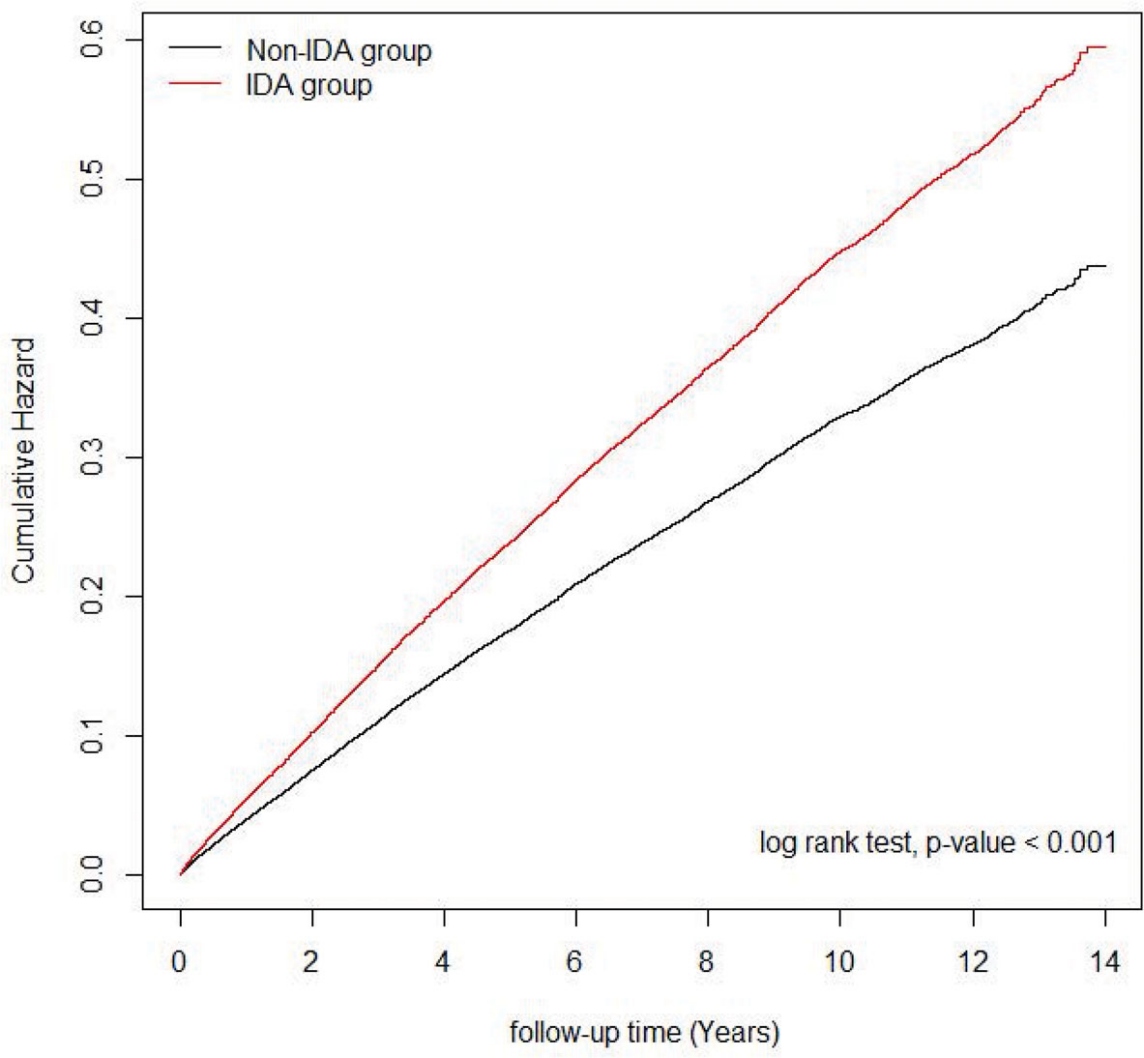
Cumulative incidences of psychiatric disorders in IDA and non-IDA groups

After adjustment for age, gender, income level, comorbidities, iron supplementation, and area, the aHR of psychiatric disorders was 1.52 (95% CI = 1.45–1.59) in the IDA group compared with the non-IDA group (Table 2). Additionally, Fig. 2 reveals that the incidence of psychiatric disorders was higher in the IDA group compared with the non-IDA group (log-rank test *p* <  0.001). A multivariate Cox proportional hazards analysis identified older age, female gender, low income, hypertension, DM, dyslipidemia, hyperthyroidism, COPD, stroke, CAD, CKD, cirrhosis, and non-iron supplementation as independent risk factors for psychiatric disorders.

**Table 2:** Univariate and multivariate analyses of risk factors for psychiatric disorders

	Crude HR (95% CI)	*p-*value	Adjusted HR (95% CI)	*p-*value
IDA	1.36 (1.31–1.41)	< 0.001	1.52 (1.45–1.59)	< 0.001
Age				
≦ 50	1.00		1.00	
> 50	1.04 (1.01–1.08)	0.017	1.04 (1.00–1.09)	0.081
Gender				
Female	1.00		1.00	
Male	0.71 (0.68–0.74)	< 0.001	0.65 (0.62–0.68)	< 0.001
Income level				
Low	1.00		1.00	
Intermediate	0.99 (0.96–1.02)	0.543	1.00 (0.96–1.03)	0.846
High	0.87 (0.81–0.93)	< 0.001	0.90 (0.84–0.97)	0.005
Comorbidity				
Hypertension	1.20 (1.15–1.25)	< 0.001	1.13 (1.07–1.19)	< 0.001
DM	1.07 (1.02–1.13)	0.013	0.88 (0.82–0.94)	< 0.001
Dyslipidemia	1.30 (1.24–1.37)	< 0.001	1.23 (1.16–1.30)	< 0.001
Hyperthyroidism	1.34 (1.21–1.49)	< 0.001	1.19 (1.07–1.33)	0.001
Hypothyroidism	1.33 (1.11–1.60)	0.002	1.11 (0.92–1.33)	0.272
COPD	1.26 (1.20–1.32)	< 0.001	1.21 (1.16–1.27)	< 0.001
Stroke	1.11 (1.04–1.19)	0.003	0.95 (0.88–1.03)	0.205
CAD	1.27 (1.21–1.34)	< 0.001	1.13 (1.06–1.21)	< 0.001
CKD	1.05 (0.93–1.18)	0.466	0.87 (0.77–0.98)	0.024
Cirrhosis	0.87 (0.74–1.03)	0.108	0.82 (0.70–0.97)	0.023
Iron supplementation	1.14 (1.10–1.18)	< 0.001	0.82 (0.78–0.86)	< 0.001
Area				
Urban	1.00		1.00	
Suburban	0.98 (0.95–1.02)	0.347	0.98 (0.94–1.02)	0.263
Rural	0.92 (0.86–0.99)	0.034	0.90 (0.84–0.97)	0.009
Remote area	0.98 (0.90–1.08)	0.689	0.96 (0.87–1.05)	0.383

*DM* Diabetes mellitus, *CAD* Coronary artery disease, *CKD* Chronic kidney disease, *COPD* Chronic Obstructive Pulmonary Disease

### Types of psychiatric disorders after IDA

Among the different types of psychiatric disorders, the IDA group was associated with significantly higher incidence and risks of anxiety disorders (aHR = 1.47, 95% CI = 1.33–1.63, *p* <  0.001), depression (aHR = 1.49, 95% CI = 1.33–1.66, *p* <  0.001), psychotic disorders (aHR = 1.41, 95% CI = 1.07–1.86, *p* <  0.050) and sleep disorders (aHR = 1.53, 95% CI = 1.46–1.61, *p* <  0.001) (Table 3). Bipolar disorder and RLS did not have a significantly higher incidence in the IDA group.

**Table 3:** Incidence, incidence rate ratio and hazard ratio of time until different type of psychiatric disorders between IDA group and non-IDA group

	IDA			Non-IDA				
Variables	Event	PY	Rate^a^	Event	PY	Rate^†^	IRR	Adjusted HR^‡^
							(95% CI)	(95% CI)
Overall	5408	118,071.60	45.80	8506	253,762.48	33.52	1.37 (1.32–1.41)^***^	1.52 (1.45–1.59)^***^
Anxiety disorders	1183	143,459.13	8.25	1739	291,222.14	5.97	1.38 (1.28–1.49)^***^	1.47 (1.33–1.63)^***^
Depression	940	144,477.03	6.51	1397	292,544.68	4.78	1.36 (1.25–1.48)^***^	1.49 (1.33–1.66)^***^
Psychotic disorders	138	148,802.23	0.93	216	298,418.90	0.72	1.28 (1.04–1.59)^*^	1.41 (1.07–1.86)^*^
Bipolar disorders	70	149,237.96	0.47	130	298,949.78	0.43	1.08 (0.81–1.44)	1.18 (0.79–1.74)
Sleep disorders	4870	121,755.17	40.00	7590	259,317.13	29.27	1.37 (1.32–1.42)^***^	1.53 (1.46–1.61)^***^
RLS	54	149,295.29	0.36	67	299,237.33	0.22	1.62 (1.13–2.31)^**^	1.30 (0.80–2.12)

*IRR* Incidence rate ratio, *PY* Person-years; ^a^Rate, incidence rate in per 1000 person-years; ^***^p <  0.001, ^**^p <  0.01, ^*^p <  0.05; ^‡^Adjusted for age, gender, income level, hypertension, DM, dyslipidemia, CAD, stroke, CKD, cirrhosis, hyperthyroidism, hypothyroidism, COPD, iron supplementation and area

### Risk of psychiatric disorders in the IDA group with or without iron supplementation

After adjusting for confounding factors, iron supplementation in IDA subjects was associated with a significantly lower risk of psychiatric disorders compared with no iron supplementation in IDA patients (aHR = 0.85, 95% CI = 0.80–0.90). Moreover, IDA patients in the iron supplementation group had a significantly lower risk of sleep disorders than IDA patients in the group without iron supplementation (aHR = 0.84, 95% CI = 0.79–0.89, *p* <  0.001, Table 4).

**Table 4:** The risk of different type of psychiatric disorders in the IDA group with or without iron supplementation

	Crude HR (95% CI)	*p-*value	Adjusted HR^a^ (95% CI)	*p-*value
Overall	0.90 (0.85–0.95)	< 0.001	0.85 (0.80–0.90)	< 0.001
Anxiety disorders	1.00 (0.89–1.13)	0.993	0.91 (0.80–1.03)	0.129
Depression	0.97 (0.85–1.11)	0.967	0.89 (0.77–1.02)	0.096
Psychotic disorders	0.88 (0.62–1.25)	0.473	0.93 (0.65–1.33)	0.686
Bipolar disorders	1.01 (0.74–1.39)	0.944	0.96 (0.57–1.61)	0.866
Sleep disorders	0.89 (0.84–0.95)	< 0.001	0.84 (0.79–0.89)	< 0.001
RLS	1.49 (1.02–2.17)	0.038	1.45 (0.99–2.14)	0.057

^a^Adjusted for age, income level, hypertension, DM, dyslipidemia, CAD, stroke, CKD, cirrhosis, hyperthyroidism, hypothyroidism, COPD, and area

#### Stratification by gender for the risk of psychiatric disorders

Table 5 displays the gender stratification analysis of the risk of IDA-associated psychiatric disorders. We demonstrate that IDA patients with or without iron supplementation had a higher risk of psychiatric disorders compared with the non-IDA group (irrespective of sex, *p* <  0.05).

**Table 5:** Adjusted HRs measured using multiple Cox proportional model for the patients with psychiatric disorders associated with IDA combines effect of iron supplementation, with stratification by gender

		Male		Female	
Iron supplementation	IDA	Adjusted HR^‡^ (95% CI)	*p-*value	Adjusted HR^‡^ (95% CI)	*p-*value
–	–	1.00		1.00	
+	–	0.75 (0.54–1.06)	0.100	0.69 (0.61–0.78)	< 0.001
–	+	1.42 (1.28–1.58)	< 0.001	1.49 (1.40–1.58)	< 0.001
+	+	1.15 (1.03–1.28)	0.017	1.28 (1.23–1.34)	< 0.001

^‡^Adjusted for age, income level, hypertension, DM, dyslipidemia, CAD, stroke, CKD, cirrhosis, hyperthyroidism, hypothyroidism, COPD, and area

## Discussion

This nationwide population-based cohort study indicated IDA as a potential risk factor for developing psychiatric disorders, even after adjusting for age, gender, income, urbanization, and comorbidities. Among the different psychiatric disorders, our results revealed that IDA was associated with an increased risk of anxiety disorders, depression, sleep disorders, and psychotic disorders. IDA with iron supplementation was associated with significantly lower risks of psychiatric disorders. Furthermore, IDA patients receiving iron supplementation had a lower risk of sleep disorders.

Our results are generally consistent with the findings of previous studies [14–16, 25, 26]. A hospital-based case-control study with 100 cases and 100 controls showed a relationship between IDA and depressive disorder; and the severity of symptoms of depressive disorder increased with the degree of IDA [15]. A web-based survey, which consisted of 1000 individuals and 10,876 controls, indicated that IDA was associated with a self-reported history of depression [26]. A case-control study which included 2957 IDA patients and 11,828 healthy controls showed a higher risk of psychiatric disorders, including mood disorders, autism spectrum disorder, attention deficit hyperactivity disorder, and developmental disorders [14]. Our study used a large population-based dataset and longitudinal design, which may have reduced surveillance bias and enabled the consideration of possible confounders for the development of psychiatric disorders. Therefore, it could be useful for explaining the causality between IDA and psychiatric disorders.

In the present study, we demonstrated that IDA was associated with an increased risk of sleep disorders. A cross-sectional study showed that IDA affects sleep quality irrespective of psychological symptoms such as depression and anxiety [16]. A possible explanation was that changes in neurotransmitter metabolism due to iron deficiency, psychological status, or possible RLS affected sleep negatively. In addition, the incidence of bipolar disorder was similar between the two groups in the present study. The following is one possible interpretation. The causes of bipolar disorder are not entirely understood. A large body of evidence has indicated that there are a number of factors working together to make a person more likely to develop bipolar disorder, such as genetics, chemical imbalances in the brain, environmental factors, physical illness, and stress [27]. It is possible that IDA only accounts for a small part of the factors affecting bipolar disorder. Therefore, we conjectured that IDA may not be the main risk factor for bipolar disorder.

Previous studies have reported the effects of iron on brain activity and mood presentation [10, 28, 29]. Iron is involved in many neurological activities and deficiency is associated with anxiety and depressive symptoms as well as developmental problems [2, 10]. In the present study, iron supplementation was shown to mitigate the risk of psychiatric disorders. We found that iron supplementation in non-IDA female subjects was associated with significantly lower risks of psychiatric disorders. Our results also found that iron supplementation has the benefit of reducing risks of sleep disorders in IDA patients. Similar to our finding, a study in Japan demonstrated that iron intake could reduce the risk of depression [26]. Another study in Korea found a negative association between depression and intake of iron after adjusting for confounding variables [30]. A meta-analysis also indicated an inverse association between dietary iron intake and risk of depression [31]. Moreover, a couple of studies also indicated that higher iron intake has a beneficial effect on lowering the risks of developing depressive symptoms [30, 32]. Several mechanisms are suggested for the relationship between iron deficiency and psychiatric disorders. Iron deficiency results in an alteration of monoamine neurotransmitters and the abnormal myelination of white matter [33, 34] . Glutamate and GABA homeostasis are modified by fluctuations in brain iron status [35]. Such alterations bring about emotional and psychological problems. Iron is essential for a number of enzymes involved in neurotransmitter synthesis, including serotonin, dopamine and norepinephrine [36], which are involved in the regulation of mood, neuronal activity, and anxiety [37, 38]. Iron deficiency is usually associated with a low level of serotonin. Previous studies have shown that serotonin deficiency may cause a relapse of depression [39, 40]. In addition, evidence has shown that impaired emotional behaviors are associated with iron deficiency via modified dopamine metabolism [39–43]. Therefore, these possible biological mechanisms may explain why iron intake could reduce the risk of psychiatric disorders.

In the present study, the development of psychiatric disorders in IDA patients with or without iron supplementation was significantly higher than the non-IDA group. These results are similar to those from research by Hong et al. [44]. They found IDA patients displayed a higher risk of Parkinson’s disease, which remained unaffected by iron supplementation [44]. This may be due to non-responsiveness to iron therapy in some patients with IDA [45]. In addition, patients with IDA, inflammation or other coexisting conditions may have reduced intestinal absorption of iron and inhibited release of iron from stores [46]. Therefore, even IDA patients receiving iron supplementation had a higher incidence of psychiatric disorders compared to the non-IDA group.

Our results are consistent with previous studies that showed a higher prevalence rate of IDA among the female population [47–49]. We observed that in the national population, among patients with IDA, the number of male patients (*n* = 4506, 23.23%) was fewer than the number of female patients (*n* = 14,891, 76.77%). A previous study demonstrated that women who have particularly heavy or prolonged menstrual bleeding, as well as pregnant and lactating women, are especially at risk of developing IDA [50]. Moreover, women with hypermenorrhea have more frequent visits to clinics or hospitals, where ICD codes are coded and diagnoses obtained accordingly, compared to men. These are possible explanations for the high prevalence rate of IDA among the female population.

### Strengths and limitations

An advantage of our study was its large sample size, which provided adequate statistical power to elucidate this important theme and also helped reduce selection bias. However, this study had several insufficiencies that should be addressed. First, some important information is not recorded in the NHIRD: psychological status, nutrition status, sleep quality, lifestyle factors, individual behavior, and family history of mental illness. These might be confounding factors. Second, the prevalence of psychiatric disorders was likely underestimated because only the subjects who used the medical resource to seek psychiatric help were identified. Finally, it would be difficult to assess the influence of iron deficiency or IDA severity on psychiatric disorder risk in this study. Subsequent studies are necessary to explain the possible relationship between psychiatric disorders and iron deficiency or IDA severity.

## Conclusions

In conclusion, our study provides epidemiological evidence that IDA may play a role in increasing the risk of psychiatric disorders. In IDA patients, iron supplementation could mitigate the risk of psychiatric disorders. Moreover, patients in the IDA group receiving iron supplementation had a lower risk of sleep disorders. Further study is necessary to explore the severity of IDA and psychiatric symptoms and to clarify the mechanisms in the association between IDA and psychiatric disorders.

## Supplementary information

**Additional file 1: Table S1.** Iron supplementation analyzed in the study

## Data Availability

The dataset used in this study is held by the Taiwan Ministry of Health and Welfare (MOHW). Any researcher interested in accessing this dataset can submit an application form to the MOWH requesting access. Please contact the staff of MOHW (Email: wt.vog.whom@uwloracts) for further assistance. Taiwan MOHW address: No. 488, Sec. 6, Zhongxiao E. Road, Nangang District, Taipei City 115, Taiwan. Phone: + 886 2 8590 6848.

## Abbreviations

AHR: Adjusted hazard ratios
CAD: Coronary artery disease
CI: Confidence interval
CKD: Chronic kidney disease
COPD: Chronic pulmonary disease
DM: Diabetes mellitus
GABA: Glutamate and γ-aminobutyric acid
HRs: Hazard ratios
ICD-9-CM: International Classification of Disease, 9th Revision, Clinical Modification
IDA: Iron deficiency anemia
LHID 2005: Longitudinal Health Insurance Database 2005
NHI: National Health Insurance
NHIRD: National Health Research Institutes
NT: New Taiwan Dollars
RLS: Restless legs syndrome
SD: Standard deviations
TIBC: Total iron binding capacity

## References

[1] Grosbois B, Decaux O, Cador B, Cazalets C, Jego P (2005). Human iron deficiency. Bull Acad Natl Med.

[2] Abbaspour N, Hurrell R, Kelishadi R (2014). Review on iron and its importance for human health. J Res Med Sci.

[3] Connor JR, Menzies SL, Burdo JR, Boyer PJ (2001). Iron and iron management proteins in neurobiology. Pediatr Neurol.

[4] Camaschella C (2015). Iron-deficiency anemia. N Engl J Med.

[5] Vukovic-Cvetkovic V, Plavec D, Lovrencic-Huzjan A, Galinovic I, Seric V, Demarin V (2010). Is iron deficiency anemia related to menstrual migraine? Post hoc analysis of an observational study evaluating clinical characteristics of patients with menstrual migraine. Acta Clin Croat.

[6] Wang J, O'Reilly B, Venkataraman R, Mysliwiec V, Mysliwiec A (2009). Efficacy of oral iron in patients with restless legs syndrome and a low-normal ferritin: a randomized, double-blind, placebo-controlled study. Sleep Med.

[7] Dhur A, Galan P, Hercberg S (1989). Iron status, immune capacity and resistance to infections. Comp Biochem Physiol A Comp Physiol.

[8] Lacey EP (1990). Broadening the perspective of pica: literature review. Public Health Rep.

[9] Todorich B, Pasquini JM, Garcia CI, Paez PM, Connor JR (2009). Oligodendrocytes and myelination: the role of iron. Glia..

[10] Kim J, Wessling-Resnick M (2014). Iron and mechanisms of emotional behavior. J Nutr Biochem.

[11] Balducci L, Ershler WB, Krantz S (2006). Anemia in the elderly-clinical findings and impact on health. Crit Rev Oncol Hematol.

[12] Haas JD, Brownlie T (2001). Iron deficiency and reduced work capacity: a critical review of the research to determine a causal relationship. J Nutr.

[13] Szajewska H, Ruszczynski M, Chmielewska A (2010). Effects of iron supplementation in nonanemic pregnant women, infants, and young children on the mental performance and psychomotor development of children: a systematic review of randomized controlled trials. Am J Clin Nutr.

[14] Chen MH, Su TP, Chen YS, Hsu JW, Huang KL, Chang WH (2013). Association between psychiatric disorders and iron deficiency anemia among children and adolescents: a nationwide population-based study. BMC Psychiatry.

[15] Shafi M, Taufiq F, Mehmood H, Afsar S, Badar A (2018). Relation between depressive disorder and Iron deficiency Anemia among adults reporting to a secondary healthcare facility: a hospital-based case control study. J Coll Physicians Surg Pak.

[16] Murat S, Ali U, Serdal K, Suleyman D, Ilknur P, Mehmet S (2015). Assessment of subjective sleep quality in iron deficiency anaemia. Afr Health Sci.

[17] Kolukisa M, Soysal P, Guletkin TO, Karatoprak C, Bilgen HR, Gursoy AE (2016). Restless leg syndrome/Willis-Ekbom disease in women with iron deficiency anemia. Ideggyogy Sz.

[18] Atkinson HH, Cesari M, Kritchevsky SB, Penninx BW, Fried LP, Guralnik JM (2005). Predictors of combined cognitive and physical decline. J Am Geriatr Soc.

[19] Yi S, Nanri A, Poudel-Tandukar K, Nonaka D, Matsushita Y, Hori A (2011). Association between serum ferritin concentrations and depressive symptoms in Japanese municipal employees. Psychiatry Res.

[20] Lever-van Milligen BA, Vogelzangs N, Smit JH, Penninx BW (2014). Hemoglobin levels in persons with depressive and/or anxiety disorders. J Psychosom Res.

[21] Cheng CL, Lee CH, Chen PS, Li YH, Lin SJ, Yang YH (2014). Validation of acute myocardial infarction cases in the national health insurance research database in Taiwan. J Epidemiol.

[22] Chou IC, Lin HC, Lin CC, Sung FC, Kao CH (2013). Tourette syndrome and risk of depression: a population-based cohort study in Taiwan. J Dev Behav Pediatr.

[23] Liang JA, Sun LM, Muo CH, Sung FC, Chang SN, Kao CH (2011). The analysis of depression and subsequent cancer risk in Taiwan. Cancer Epidemiol Biomarkers Prev.

[24] Liu C-Y, Hung Y-T, Chuang Y-L, Chen Y-J, Weng W-S, Liu J-S (2006). Incorporating development stratification of Taiwan townships into sampling design of large scale health interview survey. J Healthc Manag.

[25] Chung SD, Sheu JJ, Kao LT, Lin HC, Kang JH (2014). Dementia is associated with iron-deficiency anemia in females: a population-based study. J Neurol Sci.

[26] Hidese S, Saito K, Asano S, Kunugi H (2018). Association between iron-deficiency anemia and depression: a web-based Japanese investigation. Psychiatry Clin Neurosci.

[27] Vieta E, Berk M, Schulze TG, Carvalho AF, Suppes T, Calabrese JR (2018). Bipolar disorders. Nat Rev Dis Primers.

[28] Pino JMV, da Luz MHM, Antunes HKM, Giampa SQC, Martins VR, Lee KS (2017). Iron-restricted diet affects brain ferritin levels, Dopamine Metabolism and Cellular Prion Protein in a Region-Specific Manner. Front Mol Neurosci.

[29] Bourre JM (2006). Effects of nutrients (in food) on the structure and function of the nervous system: update on dietary requirements for brain. Part 2 : macronutrients. J Nutr Health Aging.

[30] Kim TH, Choi JY, Lee HH, Park Y (2015). Associations between dietary pattern and depression in Korean adolescent girls. J Pediatr Adolesc Gynecol.

[31] Li Z, Li B, Song X, Zhang D (2017). Dietary zinc and iron intake and risk of depression: a meta-analysis. Psychiatry Res.

[32] Miki T, Kochi T, Eguchi M, Kuwahara K, Tsuruoka H, Kurotani K (2015). Dietary intake of minerals in relation to depressive symptoms in Japanese employees: the Furukawa nutrition and health study. Nutrition..

[33] de Lima MN, Laranja DC, Caldana F, Grazziotin MM, Garcia VA, Dal-Pizzol F (2005). Selegiline protects against recognition memory impairment induced by neonatal iron treatment. Exp Neurol.

[34] Xu H, Jiang H, Xie J (2017). New insights into the crosstalk between NMDARs and Iron: implications for understanding pathology of neurological diseases. Front Mol Neurosci.

[35] Ward KL, Tkac I, Jing Y, Felt B, Beard J, Connor J (2007). Gestational and lactational iron deficiency alters the developing striatal metabolome and associated behaviors in young rats. J Nutr.

[36] Elseweidy MM, Abd El-Baky AE (2008). Effect of dietary iron overload in rat brain: oxidative stress, neurotransmitter level and serum metal ion in relation to neurodegenerative disorders. Indian J Exp Biol.

[37] Calabrese F, Molteni R, Racagni G, Riva MA (2009). Neuronal plasticity: a link between stress and mood disorders. Psychoneuroendocrinology..

[38] Ruhe HG, Mason NS, Schene AH (2007). Mood is indirectly related to serotonin, norepinephrine and dopamine levels in humans: a meta-analysis of monoamine depletion studies. Mol Psychiatry.

[39] aan het Rot M, Mathew SJ, Charney DS (2009). Neurobiological mechanisms in major depressive disorder. CMAJ..

[40] Belmaker RH, Agam G (2008). Major depressive disorder. N Engl J Med.

[41] Beard JL, Chen Q, Connor J, Jones BC (1994). Altered monamine metabolism in caudate-putamen of iron-deficient rats. Pharmacol Biochem Behav.

[42] Li Y, Kim J, Buckett PD, Bohlke M, Maher TJ, Wessling-Resnick M (2011). Severe postnatal iron deficiency alters emotional behavior and dopamine levels in the prefrontal cortex of young male rats. J Nutr.

[43] Lozoff B, Corapci F, Burden MJ, Kaciroti N, Angulo-Barroso R, Sazawal S (2007). Preschool-aged children with iron deficiency anemia show altered affect and behavior. J Nutr.

[44] Hong CT, Huang YH, Liu HY, Chiou HY, Chan L, Chien LN (2016). Newly diagnosed Anemia increases risk of Parkinson's disease: a population-based cohort study. Sci Rep.

[45] Bregman DB, Morris D, Koch TA, He A, Goodnough LT (2013). Hepcidin levels predict nonresponsiveness to oral iron therapy in patients with iron deficiency anemia. Am J Hematol.

[46] Gulec S, Anderson GJ, Collins JF (2014). Mechanistic and regulatory aspects of intestinal iron absorption. Am J Physiol Gastrointest Liver Physiol.

[47] Lee JO, Lee JH, Ahn S, Kim JW, Chang H, Kim YJ (2014). Prevalence and risk factors for iron deficiency anemia in the korean population: results of the fifth KoreaNational health and nutrition examination survey. J Korean Med Sci.

[48] Al-Alimi AA, Bashanfer S, Morish MA (2018). Prevalence of Iron Deficiency Anemia among University Students in Hodeida Province, Yemen. Anemia.

[49] Shill KB, Karmakar P, Kibria MG, Das A, Rahman MA, Hossain MS (2014). Prevalence of iron-deficiency anaemia among university students in Noakhali region, Bangladesh. J Health Popul Nutr.

[50] Levi M, Simonetti M, Marconi E, Brignoli O, Cancian M, Masotti A (2019). Gender differences in determinants of iron-deficiency anemia: a population-based study conducted in four European countries. Ann Hematol.

