# Supplementary material for: Psychiatric disorders risk in patients with iron deficiency anemia and association with iron supplementation medications: a nationwide database analysis
Source: BMC Psychiatry. 2020 May 11;20:216. doi: 10.1186/s12888-020-02621-0 (PMC7216322; doi:10.1186/s12888-020-02621-0)
Supplement: Supplementary file 1 — Additional file 1: Table S1. Iron supplementation analyzed in the study [file 12888_2020_2621_MOESM1_ESM.docx]

Supplemental Table 1. Iron supplementation analyzed in the study

| Drug classification | Component name |
| --- | --- |
| Oral form | Ferric hydroxide polymaltose, ferric oxide saccharide, ferric sodium citrate, ferrous fumarate, ferrous gluconate, ferrous sulfate, iron polysaccharide |
